# Highly Efficient Biotransformation and Production of Selenium Nanoparticles and Polysaccharides Using Potential Probiotic *Bacillus subtilis* T5

**DOI:** 10.3390/metabo12121204

**Published:** 2022-12-01

**Authors:** Yuhua Duan, Mengjun Li, Sishang Zhang, Yidan Wang, Jieya Deng, Qin Wang, Tian Yi, Xingxing Dong, Shuiyuan Cheng, Yi He, Chao Gao, Zhangqian Wang

**Affiliations:** 1 National R&D Center for Se-Rich Agricultural Products Processing, School of Modern Industry for Selenium Science and Engineering, Wuhan Polytechnic University, Wuhan 430023, China; 2 State Key Laboratory of Biocatalysis and Enzyme Engineering, School of Life Sciences, Hubei University, Wuhan 430062, China; 3 Institute of Agricultural Quality Standards and Testing Technology Research, Hubei Academy of Agricultural Sciences, Wuhan 430064, China

**Keywords:** *Bacillus subtilis*, selenium nanoparticles (SeNPs), selenium polysaccharides, α-amylase activity, probiotic potential

## Abstract

Selenium is an essential microelement required for human health. The biotransformation of selenium nanoparticles has attracted increasing attention in recent years. However, little of the literature has investigated the comprehensive evaluation of the strains for practical application and the effect on the functional properties in the existence of Se. The present study showed the selenite reduction strain *Bacillus subtilis* T5 (up to 200 mM), which could produce high yields of selenium polysaccharides and selenium nanoparticles in an economical and feasible manner. Biosynthesized selenium nanoparticles by *B. subtilis* T5 were characterized systematically using UV-vis spectroscopy, FTIR, Zeta Potential, DLS, and SEM techniques. The biosynthesized SeNPs exhibited high stability with small particle sizes. *B. subtilis* T5 also possessed a tolerance to acidic pH and bile salts, high aggregation, negative hemolytic, and superior antioxidant activity, which showed excellent probiotic potential and can be recommended as a potential candidate for the selenium biopharmaceuticals industry. Remarkably, *B. subtilis* T5 showed that the activity of α-amylase was enhanced with selenite treatment to 8.12 U/mL, 2.72-fold more than the control. The genus *Bacillus* was first reported to produce both selenium polysaccharides with extremely high Se-content (2.302 g/kg) and significantly enhance the activity to promote α-amylase with selenium treatment. Overall, *B. subtilis* T5 showed potential as a bio-factory for the biosynthesized SeNPs and organ selenium (selenium polysaccharide), providing an appealing perspective for the biopharmaceutical industry.

## 1. Introduction

Selenium is an essential trace element in the diet, required for the maintenance of health and growth, which has a wide range of pleiotropic effects, including anticancer, antioxidant, and anti-inflammatory effects [1,2]. Selenium deficiency has been reported to cause Keshan disease, Kashin–Beck disease, and even infertility [3]. The chemical form of the selenium compounds also plays an important role in the activity of selenium. Selenium exists in the environment in a variety of inorganic selenium, including selenate (SeVI), selenite (SeIV), elemental Se (Se0), selenide (SeII), and organic selenium species, such as selenoprotein and selenium polysaccharide. [4]. Compared to other inorganic forms, selenium nanoparticles (SeNPs) with small particle sizes and high surface areas have superior reactivity and excellent bioavailability [5]. It was reported that selenite inhibited liver catalase and superoxide dismutase activity in mice, but SeNPs did not affect the activity of the enzymes [6]. The study showed that SeNPs had a beneficial effect on the mice suffering from tumor development [7]. Therefore, how to obtain more SeNPs has become a research focus.

Selenium nanoparticles can be synthesized using both organic and inorganic methods. The SeNPs biosynthesized by microbes have many advantages, such as stability, safety, and low cost [8]. Nowadays, many strains which could biosynthesize SeNPs have been reported, including *Bacillus paramycoides* SP3 [9], *Bacillus amyloliquefaciens* SRB04 [10], *Providencia rettgeri* HF16-A [11], *B. subtilis* BSN313 [12], *Lactobacillus brevis* LSe [13], *Lactobacillus casei* 393 [14], and *Bifidobacterium bifidum* BGN4 [15]. Recently, SeNPs have shown anticancer and antimicrobial properties that may contribute to human health, not only as a dietary supplement but also as therapeutic agents [16]. Thus, increasing the utilization rate of SeNPs in the food and medical field needs to be taken into consideration. Advantageously, when used as a food additive, SeNPs produced by probiotics do not need to be fully purified, as the bacteria applied may be suitable for the diets of humans and animals. Moreover, some studies reported that combined probiotic and selenium supplementation may improve inflammation and oxidative stress [17,18]. Consequently, probiotics might be utilized as a suitable tool for the biosynthesis of SeNPs for further medical application.

Living non-pathogenic microbes that can be used in food or as dietary supplements for ensuring health benefits in the host are referred to as probiotics by the Food and Agriculture Organization (FAO) and World Health Organization (WHO). Probiotics are reported to include, *Enterococcus*, *Lactobacillus*, *Pediococcus*, *Streptococcus*, and Gram-positive bacteria belonging to the genus *Bacillus* [19,20]. *Bacillus* sp. was widely applied for probiotics because their key advantage was the inherent ability to form rejuvenating spores under favorable conditions. Therefore, *Bacillus* sp. is considered an excellent selenium-resistant bacterium and a capable probiotic resource for the biosynthesis of SeNPs.

Furthermore, *Bacillus* sp. are widely recognized as good industrial platform strains with the ability to produce a variety of enzymes and functional proteins, such as amylase, protease, and xylanase. [21,22,23]. α-amylase plays a significant catalytic role in the breakdown of starch into its monomeric compounds in food and clinical and medical applications [21]. However, among the known bacterial sources, α-amylase activity has not been determined in the presence of selenium. It is essential to evaluate the α-amylase activities of *Bacillus* sp. strains and explore the effect on amylase production in the presence of selenium for further application.

Polysaccharides are the most studied components of extracellular polymeric substances, which have many biological activities, such as antioxidant activity, antitumor, and antiviral activity [24]. Selenium polysaccharides are a selenium complex composed of selenium and polysaccharides, which are one of the most important forms of organic selenium [25]. In previous studies, selenium polysaccharides showed better activity than polysaccharides [26]. However, natural selenium polysaccharides are not common, as they are mostly produced by plants and fungi. Only a few bacteria can produce these compounds in adequate amounts, such as *Pseudomonas* PT-8, *Enterobacter cloacae* Z0206, and *Lactococcus lactis* [27,28,29]. Additionally, natural selenium polysaccharides generally have a selenium content, which generally ranges from 0 to 50 μg g^−1^ [30]. Researchers have recently tried to resolve the problem using different methods.

This study aims to determine the biotransformation from selenite into selenium nanoparticles and evaluate the production of selenium nanoparticles and polysaccharides by *B. subtilis* T5 treated with selenium. Purified SeNPs by *B. subtilis* T5 were characterized systematically using UV-Vis spectroscopy, FTIR, Nanoparticle size, SEM, and Zeta Potential analysis. The probiotic potential of the Se-enriched *B. subtilis* T5 to survive in physiological conditions (bile salts and low pH), to adhere to the intestine mucosa through hydrophobicity assays, and to scavenge free radical capacity, were analyzed to obtain some insights into their possible use as Se-enriched probiotic strains. Moreover, *B. subtilis* T5 also exhibited functional characteristics, including α-amylase activity and polysaccharides production. For further application, the effect of selenite on α-amylase activity and selenium polysaccharides produced by *B. subtilis* T5 were determined. The results not only enriched the medical functions of *B. subtilis* but also broadened the effective application of SeNPs and Se polysaccharides.

## 2. Materials and Methods

### 2.1. Supplies and Chemicals

Sodium selenite (Na_2_SeO_3_) was purchased from Sigma (Darmstadt, Germany), and bacterial growth medium Luria–Bertani (LB) liquid broth, including 10 g tryptophan, 5 g yeast extract, and 10 g NaCl, was purchased from SCR in Shanghai city, China. Oxgall, soluble starch, potassium persulfate (K₂S₂O₈), and 2,2′-amino-di (2-ethyl-benzothiazoline sulphonic acid-6) ammonium salt (ABTS) were provided by Macklin in Shanghai city, China.

### 2.2. High Selenite Tolerance Strain Isolation and Identification

#### 2.2.1. Screening of Selenite-Tolerant Strains

The soil samples were collected from the root soil of *Cardamine violifolia* located in Enshi (N29°50′, E109°4′), Hubei province, China, where total Se contents in the soils were between 2.86 and 4.06 mg·kg^−1^. 1 g of soil sample was suspended in 100 mL sterilized water and incubated at 37 °C (200 rpm) for 1 h. The enriched culture was serially diluted ten-fold and plated on Luria–Bertani agar (LA) broth containing 0–300 mM Na_2_SeO_3_. After culturing for an additional 24 h at 37 °C, single colonies presenting with a red color, indicating selenite reduction and Se^0^ formation, were picked and subcultured onto new plates until single colonies were obtained. Fresh overnight cultures of single colonies were harvested (8000 rpm, 5 min) and suspended in LB medium. Then the cultures were added to 10 mL LB broth and supplemented with different concentrations of Na_2_SeO_3_ (0–300 mM) incubated in a 37 °C-shaking incubator (220 rpm). After incubation at 37 °C for 24 h, the strain which exhibited the highest degree of tolerance against selenite was subsequently selected for further analyses among the isolates.

#### 2.2.2. Selenite Tolerance Strain Identification

A combined biochemical, physiological, and molecular analysis was performed to identify the isolated strain. To characterize the morphology, strain T5 was cultured in LB for 48 h, and the crystal violet staining of the bacteria was observed at ×100 for microscopic observation of cell morphology. Physiological and biochemical detection were carried out according to Bergey’s Manual of Determinative Bacteriology [31]. The 16S rDNA gene was amplified through PCR using 27F (5′-AGAGTTTGATCCTGGCTCAG-3′)/1492R (5′-TACGACTTAACCCCAATCGC-3′) primer sets [32]. Conditions for 16S rDNA gene amplification were as follows: 94 °C for 4 min, 30 cycles of denaturation at 94 °C for 1 min, annealing at 56 °C for 15 s, extension at 72 °C for 90 s, followed by a final extension at 72 °C for 10 min. The 16S rDNA gene sequence was amplified and compared to the obtained sequence in the Ezbiocloud database [33]. Moreover, the phylogenetic tree was constructed using the neighbor-joining method of MEGA version 7.0 software (bootstrap method with 1000 repeats).

Additionally, a species-specific PCR approach was applied as a supplementary PCR assay to 16S rDNA sequencing for discrimination *B. subtilis* from other members of *B. subtilis* group [34]. Two sets of primers EN1F (5′-CCAGTAGCCAAGAATGGCCAGC-3′, EN1R (5′-GGAATAATCGCCGCTTTGTGC-3′) were utilized for the PCR amplification [34]. The PCR condition was as follows: 94 °C for 4 min followed by 30 cycles of touch down program (94 °C for 30 s, 62 °C for 15 s, and 72 °C for 45 s, followed by a 0.3 °C decrease of the annealing temperature every cycle). After completion of the touch down program, the ending was with a 5 min extension at 72 °C.

### 2.3. The Growth Dynamics and Selenite Reduction Activity of the Strain T5

To determine the toxicity of selenite, the overnight cultures of strain T5 were added to each of the wells in a fresh medium (1% *v*/*v*) containing 0, 5, 25, and 50 mM selenite. The optical density at 600 nm of cultures was monitored by culturing in a microbial growth analyzer (Bioscreen C MBR., Turku, Finland) with different concentrations of selenite. Plates were incubated at 37 °C for 72 h with continuous shaking (200 rpm), and the absorbance was measured every 1 h [35].

For elucidating the growth dynamics of isolate T5 exposed to Na_2_SeO_3_, the concentration of residual selenite was quantified using inductively coupled plasma-mass spectrometry (ICP-MS) [36]. The selenite biotransformation efficiency and selenium nanoparticles formation of strain T5 were tested after incubation in LB medium supplemented with 5 mM Na_2_SeO_3_. 2 mL of the isolate. T5 culture was sampled from each flask every 12 h and centrifuged at 12,000 rpm for 15 min. After centrifugation, the residual selenite was collected in the supernatant for subsequent selenite reduction analysis, and SeNPs existed in the pellet [37]. The supernatant was microwave-assisted digestion by nitric acid. The acidic samples were diluted with ultra-pure water. The concentration of selenite was quantified using the standard curve method. The setup of the negative control was carried out using the same procedure without treatment with Na_2_SeO_3_.

The internal standard addition method was utilized for ICP-MS [38], and samples were diluted directly using a nitric acid solution. The internal standard, germanium (Ge), was used to improve the accuracy. The prepared calibration solution and samples were injected into the ICP-MS, and the Se/Ge signal ratios were measured in He mode. The signal response values of the elements and the standard internal elements were measured. The selenium content in the sample was calculated as follows: X = (C − C_1_) × V × F/m∗1000.

Where X is the content of selenium to be measured in the sample; C is the mass concentration of the measured selenium in the sample solution; C_1_ is the mass concentration of the element to be measured in the sample blank; V is the constant volume of sample digestive solution; F is the dilution ratio of the sample; m is the transferred volume of the sample; and 1000 is the conversion factor.

Meanwhile, the Se^0^ content was measured using a spectrophotometric method as described [39]. First, a calibration curve was created by measuring the intensity of red Se^0^ at 490 nm after reducing selenite solutions containing 1 to 10 μmol with 25 μmol HN_2_OH·HCl. The pellet was washed with 10 mL of 1 M NaCl three times to eliminate residual selenite. The pellets were sonicated, washed twice with 10 mL of 1 M NaCl, and then dissolved in 10 mL of 1 M Na_2_S. To separate the cells, the samples were centrifuged at 8000× *g* for 20 min. The supernatant’s absorbance was then evaluated using spectrophotometry at 490 nm.

### 2.4. Characterization of Selenium Nanoparticles by Strain T5

#### 2.4.1. Scan Electron Microscopy

For SEM observation, the bacterial culture was incubated after 24 h at 37 °C in LB medium containing 5 mM Na_2_SeO_3_ and washed with 0.9% NaCl three times. The pellets were embedded in the pre-cooled fixative solution and fixed overnight at 4 °C. Then, SEM was conducted to observe the distribution of strain T5 and SeNPs using a Hitachi SU 8010 microscope [12].

#### 2.4.2. Preparation of SeNPs

To separate selenium nanoparticles from the culture broth, strain T5 was inoculated in LB medium containing 5 mM Na_2_SeO_3_ for overnight shaking culture (200 rpm) at 37 °C. After incubation, the bacterial cultures were centrifuged at 9000 rpm for 20 min, and the pellets were ground with liquid nitrogen and treated by ultra-sonication (200 W, 20 cycles of 30 s of sonication with 30 s of rest). The suspension was filtered through a 0.22-μm filter to remove bacteria, and the filtrates were extracted with n-hexane for the removal of most lipids. The water layer was collected and centrifuged at 10,000 rpm for 10 min. To further characterize the SeNPs, the DLS, Zeta Potential Analyzer, UV-Vis, and FTIR spectroscopy analyses were carried out as previously described [37,40].

### 2.5. Evaluate the Probiotic Properties and Safety Properties of Strain T5

As per the FAO/WHO guidelines, beneficial effects of probiotic bacteria are only achievable if microorganisms are able to survive the unfavorable conditions in the gastrointestinal tract, such as low pH, high concentration of bile salts, cell surface hydrophobicity, and auto-aggregation [41]. We need to evaluate strain T5 for probiotic attributes since not all probiotic strains can survive in the digestive system. So, we selected the pH and bile salts survival, auto-aggregation, hydrophobicity, and hemolytic activity test to figure out whether the strain T5 is able to survive the unfavorable conditions in the gastrointestinal tract and apply it as a probiotic.

#### 2.5.1. Tolerance to the Low pH Condition

During fasting and after a meal, the pH value in the human stomach ranges from 1.5 to 4.5 [42]. The resistance of strain T5 to acidic conditions was evaluated based on the method described, with some modifications [43]. At first, the freshly prepared culture of strain T5 (OD_600nm_ = 0.8) was inoculated into LB broth with different pH values of 2.5 and 3.5 and incubated at 37 °C for 4 h. 1 mL of the cultures was serially diluted using sterilized water, spread on the LA plate, and incubated at 37 °C for 48 h. The viable colonies that appeared were then counted, and the survival percentage was calculated using the following equation (Equation (1)), in which pH 7.0 was applied as the control.

% Survival = (CFU mL^−1^ test / CFU mL^−1^ control) × 100 (1)

#### 2.5.2. Bile Salts Tolerance

Bile salts tolerance is considered an essential characteristic of probiotics to pass through the intestinal tract [44]. The ability of strain T5 to grow in the presence of bile salts was determined using the method described, with some modifications [45]. The bile salt solutions (0.3%, 0.6%) were prepared using Oxgall, and LB broth without Oxgall was used as the control. The freshly cultured strain T5 was consequently added to each solution (10 mL) and incubated at 37 °C for 4 h. Thereafter, viable colony counts and calculation of survival percentages were performed as previously explained.

#### 2.5.3. Cell Surface Hydrophobicity

In general, the adhesion capacity of the microorganisms is a complex multistep process, which involves both electrostatic interactions and hydrophobic forces, specific interactions between the physical and chemical characteristics of the microbial surface and intestinal mucosa [46,47]. The ability of strain T5 to adhere to hydrocarbons as a measure of hydrophobicity was determined according to the method [48]. A 3 mL freshly prepared culture of strain T5 (OD_600nm_ = 0.8) was vortexed for 2 min with two equivalent hydrocarbons (chloroform, ethyl acetate) added. The phases were allowed to separate by decantation at 37 °C for 30 min and 1 h in a clean test tube. The OD_600nm_ was measured for the removed aqueous phase. The decrease in the value of absorbance of the aqueous phase was equated with the cell surface hydrophobicity (H%), which was calculated with the given formula (Equation (2)).

(2) $$H\%=\left[\frac{A_{0}-A}{A_{0}}\right]\times 100$$

where A_0_ and A are the absorbances before and after extraction with hydrocarbons.

#### 2.5.4. Auto-Aggregation

Strain T5 cultures were centrifuged at 6000 rpm for 10 min, and the precipitates were washed three times with PBS buffer (0.1 M, pH 7.4). The absorbance (OD_600nm_) was adjusted to 0.8 ± 0.05, and then the cultures were vortexed for 30 s. Then the culture was placed into the test tubes and incubated at 37 °C. The absorbance was measured at 1, 4, and 24 h intervals [49]. The auto-aggregation rate (A%) was calculated according to the following formula (Equation (3)):

(3) $$A\%=\left[\frac{A_{0}-\mathrm{At}}{A_{0}}\right]\times 100$$

where A_0_ is the absorbance value at 0 h, and A_t_ is the absorbance value at time interval.

#### 2.5.5. Hemolytic Activity

The hemolytic activity of strain T5 was determined by using LA plate containing 5% (*w*/*v*) sheep blood, and the plates were incubated for 48 h at 37 °C based on lysis of red blood cells in the medium around the colonies. The green zones around colonies (α-hemolysis), clear zones around colonies (β-hemolysis), and no zones around colonies (γ-hemolysis) on LA blood plates. Only strains with γ-hemolysis are considered safe.

### 2.6. Antioxidant Capacity of Strain T5

To obtain cell biomass containing selenite, 1 mL of the fresh inoculums (OD_600nm_ = 0.6) were incubated (37 °C, 150 rpm) in LB broth supplemented with 5 mM selenite for 48 h (1% *v*/*v*). The produced biomass was then separated by centrifugation (8000 rpm, 10 min), and the supernatant was extracted with equivalent ethyl acetate.

Antioxidant activity of the ethyl acetate by strain T5 was evaluated using DPPH and hydroxyl scavenging assays [50]. In the DPPH and hydroxyl assays, the different concentrations of ethyl acetate (15, 30, 75, 150, 300, and 450 μg mL^−1^) were tested in separate test tubes. Different concentrations of extracts (1, 3, 5, 6, 8, 15 μg mL^−1^) were determined ABTS free radical scavenging rate according to the method reported earlier, with a slight modification [51]. The EC_50_ value is the effective concentration at which free radicals were scavenged by 50% and was obtained by interpolation from linear regression analysis.

### 2.7. The α-Amylase Activity Produced by Strain T5

Strain T5 was cultured at 37 °C for 24 h in 100 mL starch broth (1% starch, 0.5% peptone, and 1.5% yeast extract) supplemented with 5 mM Na_2_SeO_3_ after it was inoculated with 1 mL of an overnight bacterial culture (1% *v*/*v*). The cell-free supernatant obtained after centrifuging the culture broth at 10,000 rpm for 10 min was used as the crude enzyme source. The reaction mixture contained 500 μL of the crude enzyme, and 1 mL of 0.1 M phosphate buffer (pH 6.0) was added to 2 mL of soluble starch (0.5%). After incubation at 40 °C for 5 min in a water bath, the reaction was terminated using Dinitro alicyclic acid (DNS) solution (500 μL) by boiling the mixture for 5 min. The OD of the reaction mixture was measured at 540 nm. One unit of enzyme activity is defined as the amount of enzyme required to liberate one μmoL maltose per min under the assay conditions [52]. A control set without Na_2_SeO_3_ was maintained for the experiment.

### 2.8. Production of Polysaccharides by Isolate T5

#### 2.8.1. Extraction of Crude Polysaccharide

The crude polysaccharide was extracted from B. subtilis T5 after incubation for 48 h in LB medium using the following method [53]. Briefly, 10 mL fermented broth was extracted using a hot water bath. The hot-water extracts were centrifuged and precipitated by the addition of EtOH and further treated with trichloroacetic acid (TCA). The supernatant was then extensively dialyzed for 72 h (MWCO 3500 Da). The retentate was concentrated, centrifuged, and lyophilized to obtain the crude polysaccharide. The total sugar content was measured by the phenol-sulfuric-acid method [54].

#### 2.8.2. Determination of Se Content in the Polysaccharide

The Se content was determined with atomic fluorescence spectrophotometry (AFS) as the method [55]. The sample was digested with HNO_3_ for 2 h, then heated until the solution became colorless and clear, accompanied by white smoke. After cooling, 6 M HCl was added, heated, and concentrated to 2 mL, cooled to about 25 °C, and diluted into 25 mL with 6 M HCl. The Se content was detected by AFS over the exact weight of freeze-drying extracts. The crude polysaccharide was characterized by the FT-IR and UV-vis spectra.

### 2.9. Statistical Analysis

All measurements were tested in triplet for each experiment, and the results were expressed as means ± standard deviation (SD). SPSS Statistics software was used for statistical analysis. ANOVA one-way analysis of variance and Duncan’s multiple range test were used to study the significant difference between the average, where the values of *p* ≤ 0.05 were considered significant.

## 3. Results

### 3.1. Identification of Selenite-Reducing Strain

Strain T5 with high selenium tolerance and transforming ability was obtained from the isolated strains in Table S1. Moreover, the red selenium nanoparticles could be formatted in the LB agar plate containing 200 mM selenite. It was typically characterized as a rod-shaped, gram-positive bacterium by morphological observations, and the colony formed was gray-white, opaque, and drying, with thick ridges on the LB agar plate. Strain T5 was associated with the reduction of selenite to SeNPs and the appearance of orange to dark red colonies in the LA plate containing 200 mM selenite (Figure S1).

**Figure 1:**
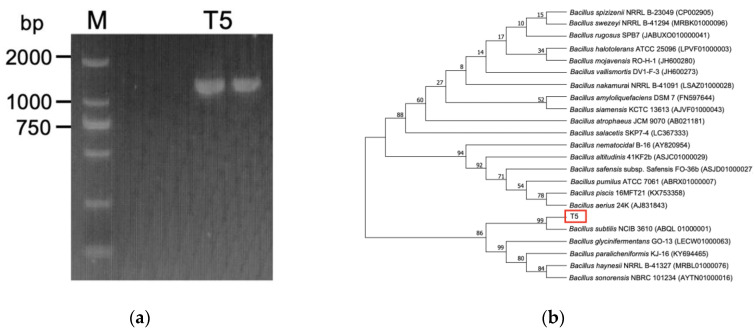
Identification of strain T5. (**a**) 1 311 bp PCR product in strain T5 with primer combinations specific oligonucleotide primers; (**b**) The neighbor-joining phylogenetic tree based on the 16S rDNA gene sequence of strain T5.

Molecular identification based on the 16S rDNA gene sequence showed that strain T5 was closely related to show 99% 16S rDNA gene sequence similarity to *B. subtilis* NCIB 3610 (ABQL 01000001). To distinguish *B. subtilis* from other members of the *B. subtilis* group, 16S rDNA sequencing results were supplemented by PCR detection, which showed positive for strain T5 in a reproducible amplification of 1311 bp product with primer combinations of specific primers from the endoglucanase gene. The neighbor-joining phylogenetic tree also revealed that strain T5 was closely related to *B. subtilis* NCIB 3610 (ABQL 01000001) (bootstrap method with 1000 repeats) (Figure 1). The physiological and biochemical tests revealed that strain T5 showed the typical characteristics of *B. subtilis*, as indicated in Table S2. In conclusion, strain T5 was identified as *B. subtilis*.

### 3.2. Selenite Biotransformation Assays

**Figure 2:**
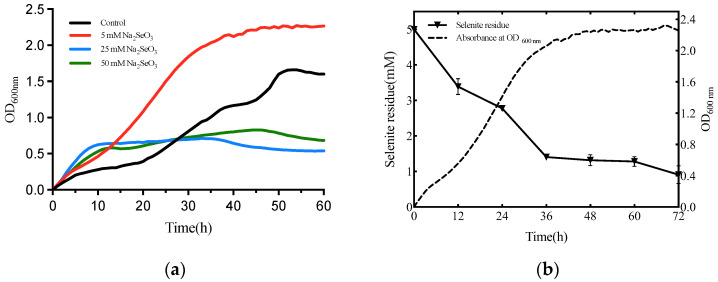
Growth curve, selenite reduction of *B. subtilis* T5 in the presence of selenite (5 mM). (**a**) Growth curve of strain T5 in the different concentrations of selenite (0, 5, 25, 50 mM); (**b**) Selenite reduction of *B. subtilis* T5 in the presence of selenite (5 mM).

The growth kinetics of *B. subtilis* T5 in the presence of selenite (0, 5, 25, 50 mM) is shown in Figure 2. The results showed that the concentration of 5 mM selenite could accelerate the growth of *B. subtilis* T5 and advance to enter a period of the exponential phase. Thus, the 5 mM selenite was chosen as the optimal concentration to further study strain T5. With the increasing concentration of selenite, the growth of strain T5 was inhibited. As indicated by ICP-MS, about 73% of the selenite was exhausted when the strain growth accessed the stationary phase (at 36 h of incubation). Remarkably, as shown in Figure 2, the reduction of selenite occurred simultaneously at the beginning of the strain growth.

### 3.3. Characterization of SeNPs by B. subtilis T5

#### 3.3.1. The DLS, Zeta Potential, and SEM Analyses Revealed the Shape and Stably of SeNPs

**Figure 3:**
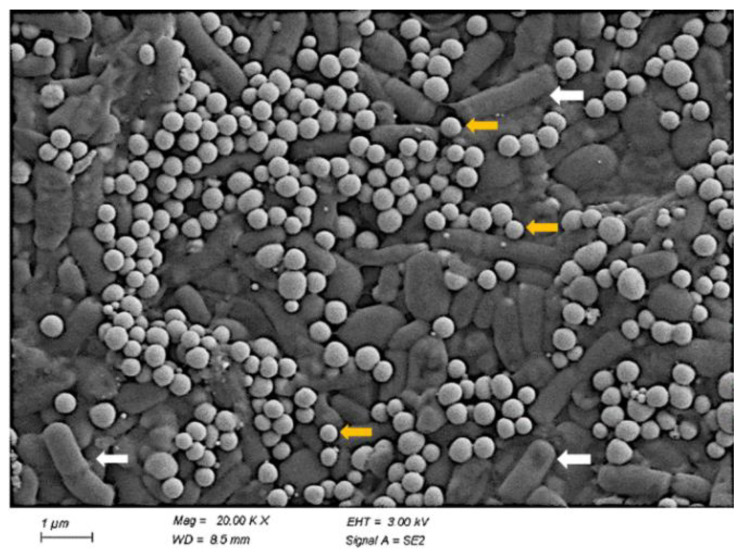
The SEM image of SeNPs (Yellow arrows) produced by *B. subtilis* T5 and the bacteria cell (White arrows).

**Figure 4:**
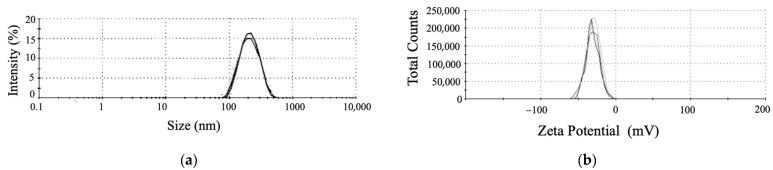
The characteristics of SeNPs produced by *B*. *subtilis* T5. (**a**) Particle size distribution; (**b**) Zeta Potential.

To clarify the effect of selenite reduction and SeNPs production on the morphology of strain T5, it was inoculated into an LB medium containing 5 mM Na_2_SeO_3_. SEM analysis showed the morphology of *B. subtilis* T5 and SeNPs after incubation for 48 h in a Se-supplied medium (Figure 3). The SEM result showed that spherical SeNPs of 100 to 200 nm were located inside bacterial cells, with a uniform shape and good dispersion. Furthermore, the integrity and shape of bacterial cells were maintained. The purified SeNPs were detected by DLS and Zeta Potential analysis in an aqueous solution (Figure 4). The DLS analysis also revealed a mean particle size of 167.8 ± 1.72 nm. The Zeta Potential result showed that purified SeNPs of zeta potential of −31.1 mV were stable. Each experiment was repeated at least three times. The size of the SeNPs is an important factor in determining the chemical properties and biological activities. These results reveal that *B. subtilis* T5 can produce SeNPs of small sizes and high stability, which constitutes a versatile platform that may be suitable for biotechnological applications.

#### 3.3.2. The UV-Vis and FTIR Spectrums of SeNPs

**Figure 5:**
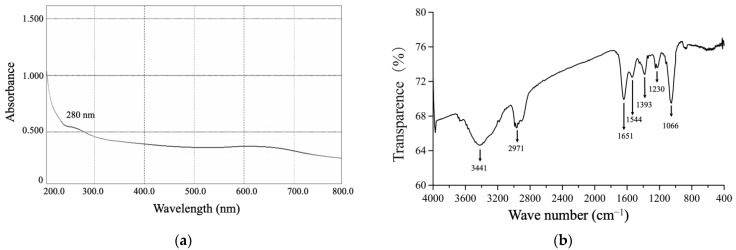
The spectra analysis of SeNPs produced by *B*. *subtilis* T5. (**a**) The UV-vis spectra; (**b**) The FTIR spectra.

The UV-Vis spectrum of the purified SeNPs exhibited a peak at 280 nm, usually attributed to aromatic amino acids, indicating the presence of proteinaceous material adhered to the surface of SeNPs (Figure 5). The BioSeNPs had a broad feature at 3441 cm^−1^ corresponding to N-H and O-H stretching vibrations of amine and hydroxyl groups of the carboxylate group in the amino acids [56]. Additionally, features between 2872 to 2989 cm^−1^ were observed, which could be attributed to aliphatic saturated C-H stretching modes in protein side chains [57]. The amide I and amide II linkages could be recognized at observed bands at 1651 and 1544 cm^−1^, which corresponded to the vibrations of carbonyl and N–H stretches in the amide linkages of the proteins, respectively [57]. The feature at 1395 cm^−1^ could be attributed to C=O of COO^−^ symmetric stretching in proteins [58]. The strongest peak observed at 1230 cm^−1^ could be attributed to the C–N stretching and N–H bending vibrations of proteins (amide III). The presence of carbohydrates could be evidenced by the peak observed at 1066 cm^−1^ from the C–O–C stretch of carboxylic acids and ether groups [59]. FTIR spectra for BioSeNPs confirmed the presence of proteins (amide I: 1651 cm^−1^, amide II: 1544 cm^−1^, and amide III: 1230 cm^−1^) and carbohydrates (1066 cm^−1^).

### 3.4. Probiotic Properties of B. subtilis T5

#### 3.4.1. Tolerance to Low pH and Bile Salts

The survival rate of *B. subtilis* T5 at pH 3.5 was 70.63 ± 2.07%, which was close to the survival percent at pH 2.5. Our results illustrated that the survival rate was reduced to 51.12 ± 1.67% and 46.24 ± 2.58% after 4 h of incubation in the presence of 0.3% and 0.6% of bile salts, respectively (Table 1).

#### 3.4.2. Hydrophobicity and Auto-Aggregation Properties

In general, hydrophobicity and auto-aggregation assays were utilized as tools to detect the capacity of adhesion to the intestinal mucosa. As shown in Table 1, *B. subtilis* T5 showed a high auto-aggregation percentage (80.47 ± 0.83%) after decantation for 24 h, and the hydrophobicity was determined to be 37.99 ± 2.57%.

#### 3.4.3. Hemolytic Activity

**Figure 6:**
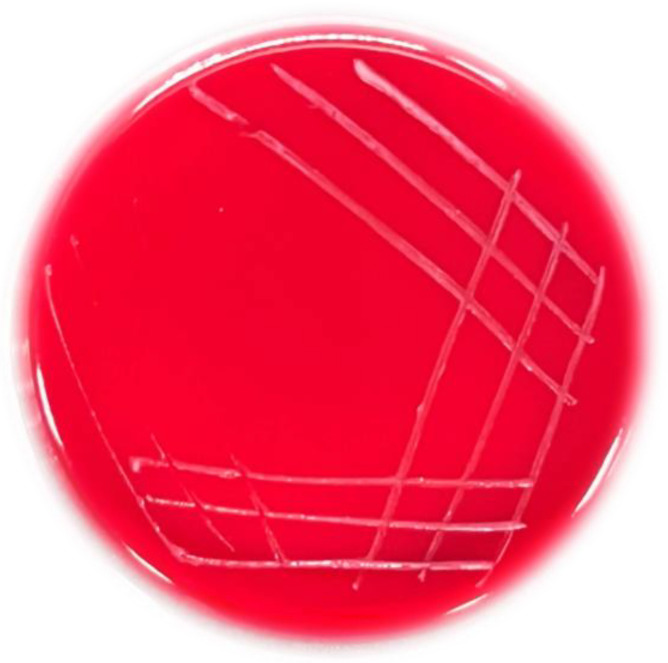
Images of cultures of *B. subtilis* T5 grown on the blood agar plate after 48 h.

The green zones around colonies (α-hemolysis), clear zones around colonies (β-hemolysis), and no zones around colonies (γ-hemolysis) on LA blood plates. Only strains with γ-hemolysis are considered safe. The hemolytic activity of strain T5 was evaluated on blood agar plates after 48 h. The strain T5 showed no hemolytic activity (Figure 6), which could be utilized for the probiotic candidate.

#### 3.4.4. Antioxidant Activities of Ethyl Acetate Extract of T5

**Figure 7:**
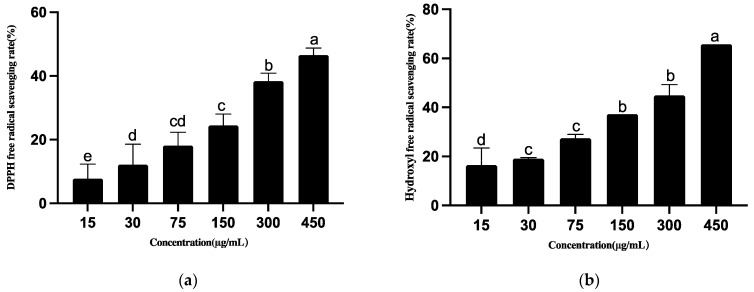

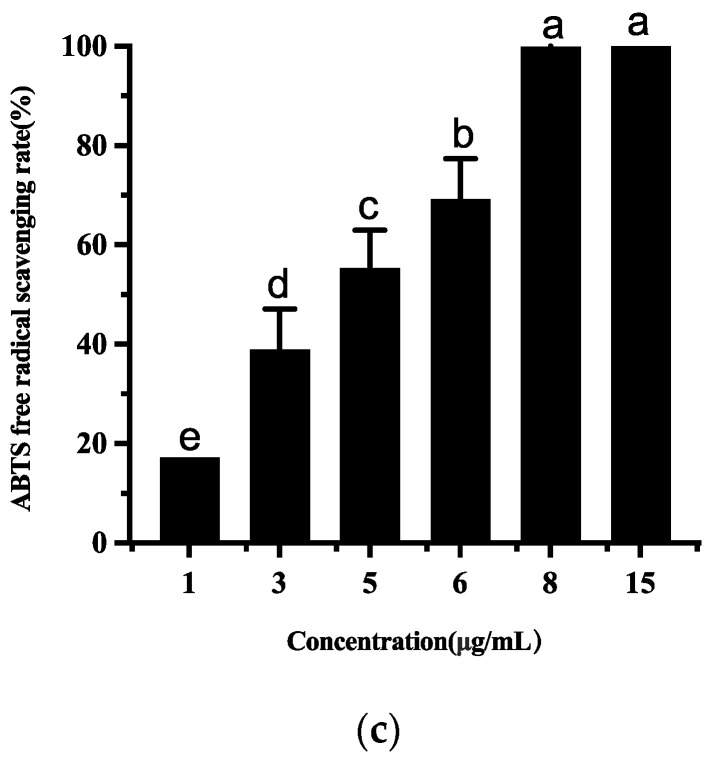
The antioxidant capacity of ethyl acetate extracts of *B. subtilis* T5. (**a**) DPPH radical scavenging activity; (**b**) Hydroxyl radical scavenging activity; (**c**) ABTS radical scavenging activity. The data were analyzed using one-way analysis of variance (ANOVA, *p* < 0.05). Different lowercase letters above the bars indicate significant differences (*p* < 0.05).

As shown in Figure 7, the ABTS radical scavenging activity of ethyl acetate extracted by *B. subtilis* T5 showed extreme antioxidant activity (99.59 ± 0.16) at the minimum concentration of 15 μg mL^−1^. Therefore, the concentration of the sample was decreased, and the resulting ABTS radical scavenging test revealed the EC_50_ of 5.729 μg mL^−1^ (Figure 7). ABTS radical scavenging activity exhibited a dose-dependent manner in the concentration range of 1–8 μg mL^−1^. The acetate extracts of *B. subtilis* T5 exhibited a maximum scavenging capacity of 65.79% against hydroxyl radicals. *B. subtilis* T5 exhibited the highest percentage of DPPH reduction (46.56 ± 2.24).

### 3.5. Functional Activity of B. subtilis T5

#### 3.5.1. α-Amylase Activity

**Figure 8:**
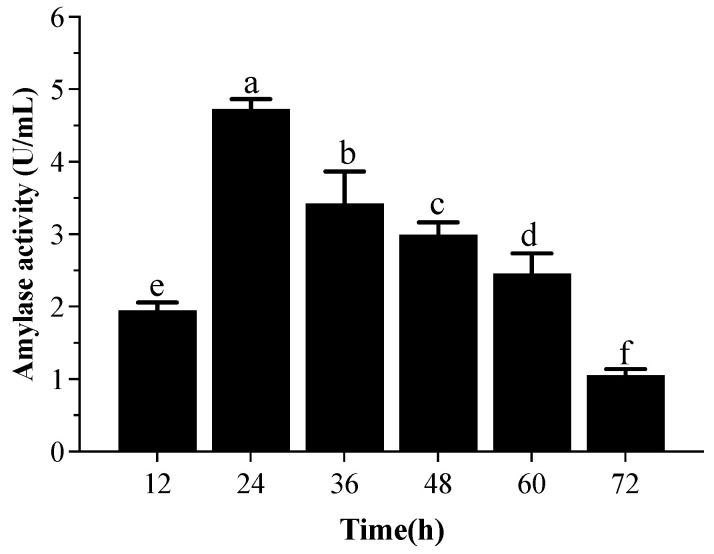
α-amylase activity by *B. subtilis* T5 with respect to different incubation periods; mean values with the different letters are significant (*p* ≤ 0.05).

The amylase production by strain T5 was observed every 12 h of incubation in Figure 8, and the optimum duration for the maximum amylase production from *B. subtilis* T5 was determined after 24 h (4.73 U/mL). In this study, *B. subtilis* T5 was shown to have potential as a tool for producing α-amylase.

#### 3.5.2. Assessment of Crude Polysaccharides

**Figure 9:**
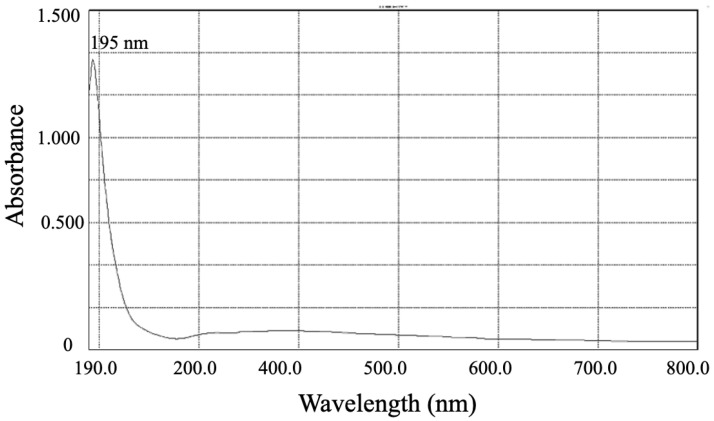
The UV-vis spectra of polysaccharides produced by strain T5.

Strain T5 was cultured by liquid fermentation after 48 h, and the yield of total lyophilized polysaccharide by *B. subtilis* T5 was 128.74 mg L^−1^. The electronic spectrum of the polysaccharide (Figure 9) showed no characteristic absorption of protein peak at around 250–300 nm of polysaccharide, indicating a relatively pure polysaccharide with few proteins.

### 3.6. Effects of Selenite on Functional Activity by B. subtilis T5

#### 3.6.1. Effects of Selenite on α-Amylase Activity by B. subtilis T5

**Figure 10:**
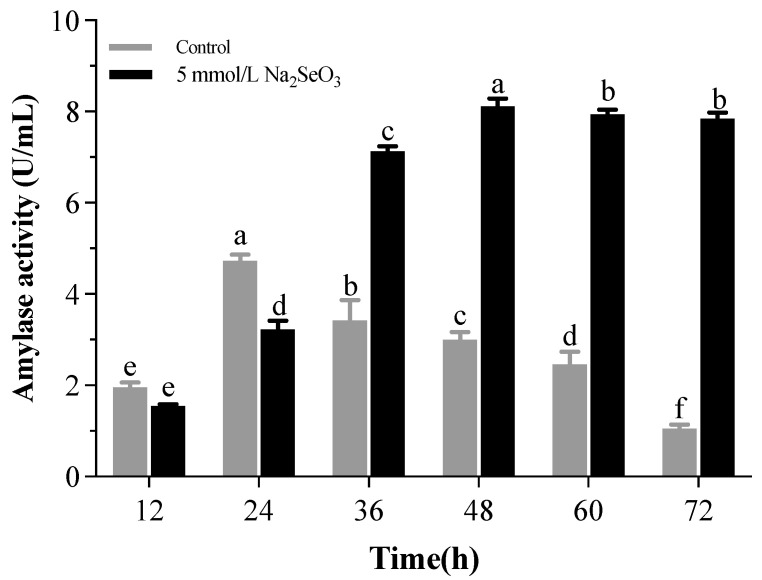
Effect of selenite on α-amylase activity by *B. subtilis* T5 with respect to different incubation periods; mean values with the different letters are significant (*p* ≤ 0.05).

The growth of strain T5 was impacted in the presence of selenite. Therefore, the amylase activity was observed by adding 5 mM selenite, which could promote the growth of strain T5. Remarkably, *B. subtilis* T5 showed that the activity of α-amylase was enhanced 2.72-fold with selenite treatment to 8.12 U/mL than the control after 48 h (Figure 10).

#### 3.6.2. Assessment of the Selenium Polysaccharides in the Presence of 5 mM Selenite

Biosynthesis of selenium-enriched polysaccharide was produced by liquid fermentation of selenium-tolerant strain T5 containing the optimal concentration of sodium selenite. There were great differences between the treatments in the presence of selenite and the control in the absence of selenite in the average polysaccharide yield and selenite content. The total polysaccharide content of strain T5 decreased to 78.15 mg L^−1^. The contents of Se in polysaccharides were 2.302 g kg^−1^ evaluated by HG-AFS. Therefore, strain T5 was a new strain that could produce the polysaccharide with high Se content in the presence of 5 mM selenite.

**Figure 11:**
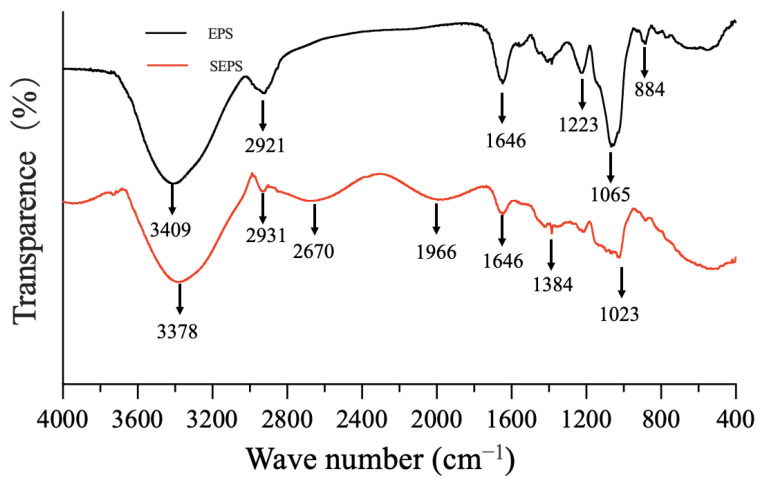
The FTIR of EPS and SEPS by *B. subtilis* T5.

Compared with polysaccharides, the absorption peak at 1023 cm^−1^ (Figure 11) is the O-Se-O bond of selenium ester [60]. This information signified that SEPS produced by *B. subtilis* T5 was successfully modified after Se-enrichment. In general, polysaccharides produced by *B. subtilis* T5 could modify the surface structure of SeNPs, which showed selenite reduction activity in the localization of selenite reduction, and selenium polysaccharide was detected in the presentence of selenite.

## 4. Discussion

Screening strains with high selenium tolerance has broad prospects, which can bio-transform toxic selenite into selenium nanoparticles. The selenite-reducing ability of strains can be evaluated by preliminarily testing the tolerance to different concentrations of selenite. In previous studies, the selenite tolerance of strains generally ranges from 20 to 150 mM. For example, *Alcaligenes faecalis* Se03 from gut samples of *Monochamus alternatus* was found to show a strong tolerance to selenite (up to 120 mM) [37]. The minimum inhibitory concentration of *Rahnella aquatilis* HX2 to selenite was 85 mM [61]. *Vibrio natriegens* were able to grow with a significant survival rate at concentrations as high as 100 mM selenite [62]. Compared to reported selenite tolerance strains, strain T5 showed a high selenium tolerance, and it could format the red SeNPs in the broth containing 200 mM selenite. *B. subtilis* T5 could be unitized as a candidate for biosynthesis SeNPs for nutritional supplementation due to the high selenite tolerance.

The selenite oxyanion has already been widely recognized to have toxic effects on bacterial growth [35,58]. Indeed, selenite may react with thiol groups of proteins to produce free radicals, which causes severe damage to the cells [63]. In previous studies, 5 mM selenite had a toxic effect on the growth of most reported strains, such as *Pseudomonas putida* KT2440 [35], *Burkholderia fungorum* DBT1 [39]. However, the growth of strain T5 was promoted in the existence of 5 mM selenite compared to the control group. Remarkably, the reduction of selenite was observed at the very beginning of the strain T5 growth phase, confirming that the selenite concentration decreased sharply with the onset of strain growth. This phenomenon is different from most of the previously reported strains with significant selenite reduction ability, which showed the reduction of selenite in the middle of the exponential or stationary growth period [35,39,58]. Strain T5 was also able to reduce the selenite concentration of about 692 μg mL^−1^ after 72 h. Our results showed that *B. subtilis* T5 could produce selenium polysaccharides (organic selenium), not just SeNPs, in the existence of 5 mM selenite. Moreover, it may be attributed to other forms of selenium, such as hydrogen selenide (H_2_Se), that exist in a gaseous form [64]. Following this, we speculated that some forms of inorganic selenium or organic selenium might be accumulated in addition to the formation of SeNPs. Overall, strain T5 is not only a bacterial strain highly resistant to selenite but also a strain with rapid selenite reduction, which could be used as a biocatalyst for transforming selenite into SeNPs.

Previous studies have indicated that selenium polysaccharides exhibit better bioactivities, including immunomodulation, antitumor, antioxidation, and glucose regulation, than polysaccharides alone [30]. At present, fungi, algae, and plants are the most common source for preparing selenium polysaccharides [65]. Little research has been reported on the selenium polysaccharide biosynthesized by *B. subtilis* [66]. Remarkably, *B. subtilis* T5 could absorb and bio-transform selenite into selenium polysaccharides during the growth process. The Se content in the Se-EPS produced by strain T5 is at a high level of 2301.57 μg g^−1^, which is much higher than most natural selenium polysaccharides [30].

The size of nanoparticles plays an important role in biological activity. Generally, smaller-sized nanoparticles are more active than larger ones [67,68]. Most biogenic SeNPs were reported at the range of 100–500 nm [12,62]. The DLS analysis revealed the average size of SeNPs produced was 167.8 nm. Our study showed that SeNPs biosynthesized via *B. subtilis* T5 were highly stable, with a zeta potential of −31.1 mV, due to its organic capping with proteins, as revealed by UV-Vis and FTIR analyses. Micrographs showed that SeNPs were accumulated in large quantities outside the cells attached to the culture. The particles were spherical, uniform in shape, and well dispersed. Moreover, no cell lysis or shape change was observed, indicating that 5 mM sodium selenite would not affect the growth of strain T5 (Figure 3).

Survival rate at low pH and bile salts is considered an important criterion for probiotic strains to exert their beneficial effects on the human stomach [69]. *B. subtilis* T5 exhibited moderate tolerance to low pH and bile salts, which could survive in the gastrointestinal tract. *B. subtilis* T5 also had bile tolerance, but a high concentration of bile could affect the survival of strain T5, which could damage the cell membrane structure. *B. subtilis* T5 showed a high auto-aggregation and hydrophobicity percentage after decantation for 24 h. The present study has shown that *B. subtilis* T5 could adhere to intestinal mucosa well.

Oxidative stress refers to elevated intracellular levels of oxygen free radicals, which could cause damage to lipids, proteins, and DNA [70]. In recent years, the search for safer and more natural antioxidants from biological resources has attracted great attention. In a previous study, ABTS•+ scavenging ability of cell-free extracts from *Bacillus licheniformis* KT921419 ranged from 17.60 to 55.81 and 13.46 to 59.95%, respectively [71]. Our results showed that the free radical scavenging ability of ethyl acetate extracts of strain T5 showed better antioxidant activities.

The production of enzymes was influenced by the growth of strain T5 and the composition of the medium [72]. It is interesting that the selenite supplement to produce amylase by *B. subtilis* T5 resulted in higher amylase activity, which with selenite treatment, was enhanced 2.72-fold more than the control after 48 h. Many trace elements, such as Mg^+2^, Cu^+2^, Zn^+2^, and Fe^+2^ ions, have been reported to improve the production of amylase activities by strains [72,73]. The possible reason was that the activity of amylase enhanced with the increasing concentration of these elements. The enhancement of enzyme activity may have been due to the high amount of acidic amino acids on the surface of this enzyme. Negative charges on the halophilic protein bind significant amounts of hydrated ions and produce hydrated ion networks, thus reducing its surface hydrophobicity, enhancing protein solubility, and decreasing the tendency to aggregate at a high salt concentration [74]. We speculated that the addition of selenium element increased the growth state of the strain, especially as upregulating the expression of starch-related catabolic enzymes leads to a rise in sugar, accompanied by an increase in cells breathing to meet the energy needs for faster growth, thereby increasing the amylase activity. To our best knowledge, none of the literature has been published about the effects of selenium on amylase activity produced by bacteria. In addition, the enzyme production capacity is expected to be greatly improved after optimizing the induction conditions and the applications of additives, which are more suitable for industrial production.

## 5. Conclusions

In our study, *B. subtilis* T5 isolated from selenium-rich areas showed high tolerance to selenite, which shows potential for producing more selenium nanoparticles. The strain could reduce toxic selenite into SeNPs and selenium polysaccharides simultaneously at the beginning of the strain growth. Selenium nanoparticles biosynthesized by strain T5 had a small particle size. Biosynthesized SeNPs produced by *B. subtilis* T5 are stable in an aqueous solution, which has a natural coating of the organic capping. Moreover, natural selenium polysaccharides by strain T5 had extremely high Se-content. The strain *B. subtilis* T5 was also able to tolerate the simulated gastrointestinal conditions, including resistance to acidic pH, bile salts, and excellent antioxidant ability. In addition, *B. subtilis* T5 exhibitedα-amylase producing capability in the absence and presence of selenite, which is the first study on the promoting effect of selenite on α-amylase activities among the bacteria. The potential health benefit of *B. subtilis* T5 and selenium co-supplementation will further be applied as a promising probiotic strain in the fields of medicine and biology.

## Figures and Tables

**Table 1:** Probiotic characteristics of *B*. *subtilis* T5.

Probiotic Characteristics	Condition	Degree (%)
Survival tests:		
Different pH levels (for 4 h)	pH 2.5 pH 3.5	62.75 ± 1.22 70.63 ± 2.07
Different concentrations of bile salts (for 4 h)	0.3% 0.6%	51.12 ± 1.67 46.24 ± 2.58
Auto-aggregation	1 h 4 h 24 h	5.49 ± 0.57 13.59 ± 1.37 80.47 ± 0.83
Cell surface hydrophobicity	0.5 h 1 h	21.63 ± 3.44 37.99 ± 2.57

## Data Availability

The data is available on request. The data are not publicly available due to its privacy.

## Supplementary Materials

The following supporting information can be downloaded at: https://www.mdpi.com/article/10.3390/metabo12121204/s1, Figure S1: Images of cultures of strain T5 grown. (a) In the absence; (b) In the presence of 200 mM selenite; Table S1: Color changes of isolates in LA broth containing different concentrations of selenite; Table S2: Biochemical characteristics of strain T5 and *Bacillus subtilis*. Reference [31] are cited in the Supplementary Materials.
