# Supplementary material for: Highly Efficient Biotransformation and Production of Selenium Nanoparticles and Polysaccharides Using Potential Probiotic Bacillus subtilis T5
Source: Metabolites. 2022 Dec 1;12(12):1204. doi: 10.3390/metabo12121204 (PMC9784637; doi:10.3390/metabo12121204)
Supplement: Supplementary file 1 [file metabolites-12-01204-s001.zip › metabolites-2017587-supplementary.pdf]

metabolites

MDPI

Highly efficient biotransformation and production of selenium nanoparticles and polysaccharides using potential probiotic *Bacillus subtilis* T5

Supplementary Materials:

Table S1. Color changes of isolates in LA broth containing different concentrations of selenite.

Bacteria	50 mM	100 mM	150 mM	200 mM
Bacillus licheniformis ES-1	+++	++	-	-
Bacillus tequilensis ES-2	++	-	-	-
Lysinibacillus fusiformis XF-1	++++	++++	++	-
Bacillus paralicheniformis XF-4	++++	+++	++	-
Bacillus subtilis T5	++++	++++	+++	+++
Bacillus cereus T4	++++	+++	+	-
Bacillus pumilus H4T-9	+++	++	-	-

− normal; + slightly red; ++ orange; +++ red; ++++ crimson.

Table S2. Biochemical characteristics of strain T5 and *Bacillus subtilis*

Characteristic	Strain T5	*Bacillus subtilis* [1]
Oxidase	+	V
Voges–Proskauer test	—	—
Indole production	—	—
Gelatin liquefaction	V	+
Carbon sources utilization	—	—
D-xylose	—	—
L-arabinose	—	—
D-mannose	—	—
Sucrose	—	—
α-D-glucose	—	—
Lactose	—	—
Arginine decomposition	—	—
Lysine decomposition	—	—
Ornithine decomposition	—	—

+ Positive; − Negative; V variable.

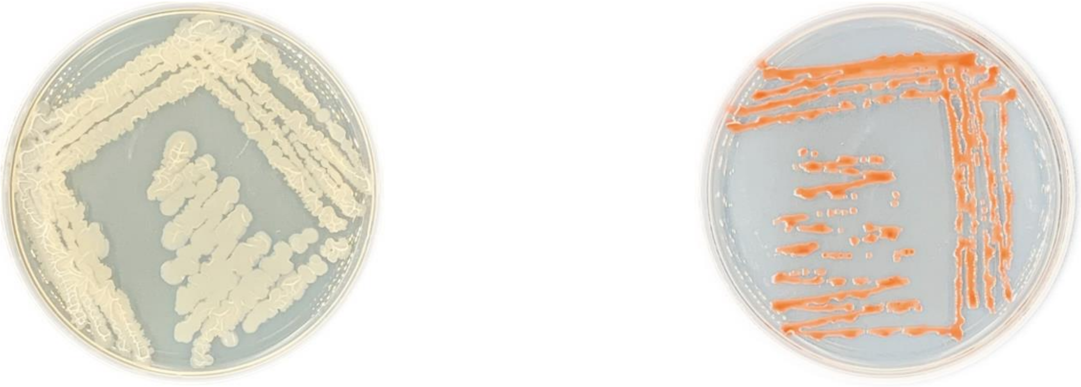

(**a**) (**b**)

Figure S1. Images of cultures of strain T5 grown. (**a**) In the absence; (**b**) In the presence of 200 mM selenite.

References:

1. N.R.K. J.G. Holt, P.H.A. Sneath, J.T. Staley, S.T. Williams, Bergey's Manual of Determinative Bacteriology. 9th ed, Baltimore: Williams and Wilkins, **1994**, pp. 255-60.
